# The impulse response of the organ of Corti at the apex of the intact cochlea

**DOI:** 10.1038/s41598-025-21860-3

**Published:** 2025-10-30

**Authors:** Alberto Recio-Spinoso

**Affiliations:** Caruso Department of Otolaryngology, University of Southern California, Los Angeles, CA 90033 USA

**Keywords:** Cochlea, Optical coherence tomography, Signal processing, Systems identification, Cochlea, Neurophysiology

## Abstract

Georg von Békésy’s initial observations of inner-ear vibrations were carried out at the apex of human-cadaver cochleae but contemporary studies have focused on responses at the base of the cochlea of live rodents. Apical cochlear recordings have proven difficult to achieve because opening the otic capsule often produces surgical injury and experimental artifacts. Using optical coherence tomography, I recorded mechanical responses to click stimuli at the organ of Corti (OoC) in the intact chinchilla cochlea at sites with characteristic frequencies (CFs) around 500 Hz. In general, OoC velocity responses to clicks consist of two segments: an initial component with a band-pass tuning centered at around 500 Hz, and a later component with low-pass characteristics. Spectral analysis of the transient responses revealed that responses had greater amplitudes and wider frequency bandwidth near the Hensen’s cells region than in the proximity of the basilar membrane, their frequency selectivity increased with stimulus level and death, and CF changes were negligible postmortem. Noise analysis, using Wiener kernels, indicates that click responses contain nonlinearities absent in first-order Wiener kernels. Click responses were well approximated by a frequency independent delay followed by a minimum-phase filter. The average length of the delay was approximately 1.1 ms.

## Introduction

The use of acoustic clicks, or sounds of brief duration, to estimate the transient response of the basilar membrane (BM) in the cochlea of laboratory species has been well documented (e.g., squirrel monkeys^1^, guinea pigs^2^, and chinchillas^3,4^, among others). With a few exceptions, such as the pioneer work of Robles and colleagues^1^, data from most of those publications originated from the basal end of the cochlea, a region sensitive to high-frequency sounds.

BM and tectorial membrane (TM) responses to clicks at the apex of the chinchilla and guinea pig cochleae were reported by Cooper and Rhode^5^. In those experiments, reflecting beads were dropped into the scala media after a cochleostomy and tearing of Reissner’s membrane. The responses to clicks consisted of distinct “fast” and “slow” components. Because the amplitude of the fast response diminished significantly after carefully sealing the otic capsule with a glass cover slip, it was concluded that the fast response component was likely artifactual^5,6^.

The application of optical coherence tomography (OCT)^7^ has made it possible to record sound-evoked vibrations at the apex of the cochlea without the need for a cochleostomy^8–10^. OCT also provides a means to test the hypothesis that fast responses are absent at the apex of the intact cochlea. In this report, I describe the transient response of the organ of Corti (OoC) at the apex of the intact chinchilla cochlea obtained using a commercially available OCT system. OoC velocity responses to clicks consist of two segments with distinct properties: the initial component is a band-pass signal tuned to the characteristic frequency (CF: frequency of greatest sensitivity in a normal cochlea) of the recording site and it is followed by a low-pass component. Vibratory responses to exponential-sine signals^11,12^ and Gaussian noise ^13,14^ were also measured at sites with a CF of around 500 Hz, as in^5^.

Apical OoC responses to clicks at a site with CF≈500 Hz in the intact cochlea have an onset delay (relative to the onset of middle-ear motion) averaging 1.1 ms. Responses to clicks at this cochlear location can be well approximated by an onset delay followed by a minimum-phase filter. These responses exhibit compressive nonlinearities which differ from those of BM responses at the base of the chinchilla cochlea^3,4^.

## Results

Adult chinchillas (N = 8) of either sex weighing around 500 g were used. Experiments reported here were part of a larger cohort involving a study of the OoC responses to single tones at the apex of the chinchilla cochlea^15^. CFs, determined using 1-s tones presented 2 or 3 times, were between 400 and 500 Hz.

### Basic properties of organ of Corti (OoC) responses to clicks at the apex of the cochlea

**Fig. 1:**
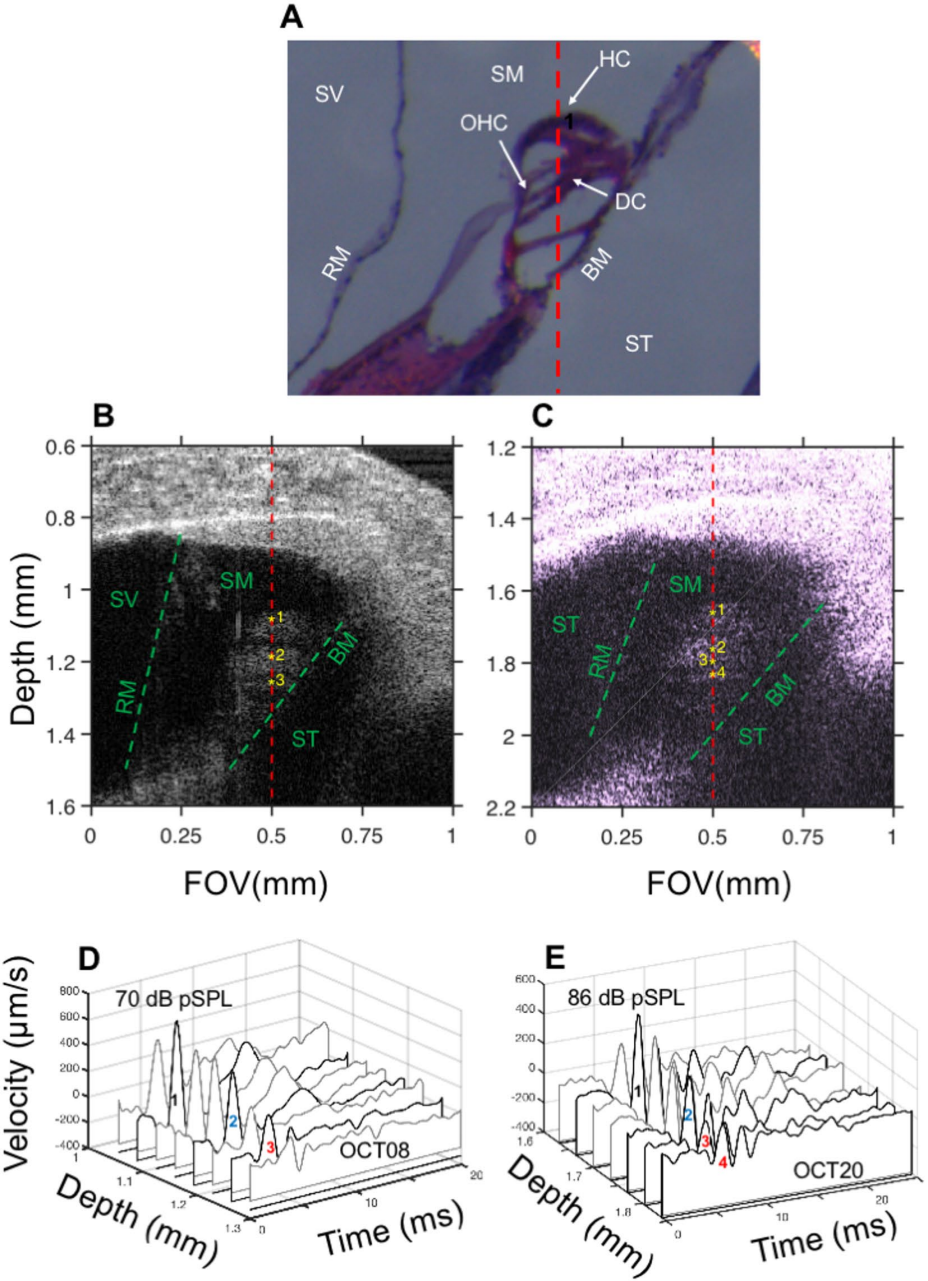
Imaging and vibrometry of the intact chinchilla cochlea. Panel (**A**) shows a histological cross-section of a cochlea stained with hematoxylin and eosin. Labels indicate: SV: scala vestibuli; SM: scala media; ST: scala tympani; RM: Reissner’s membrane; BM: basilar membrane; OHC: outer hair cells; HC: Hensen’s cells; DC: Deiters’ cells. Panels (**B**) and (**C**) display cross-sections (B-scans) of the apical ends of two right cochleae (OCT08 and OCT20, respectively). Several cochlear structures are discernible in the B-scan: SV, ST, SM, RM and BM. Waterfall plots in D and E display organ of Corti responses to 70 dB pSPL and 86 dB pSPL clicks, respectively, at several locations along the optical axis (red dashed lines in B and C). Numbers in the waterfall plot indicate the locations of the responses in the B-scans (1–3 in D; 1–4 in E). Waveforms at locations 1–3 in D and 1–4 in E are plotted using black lines; the remaining waveforms are depicted using gray lines.

Previous studies have established that responses to single tones or tone complexes differ among different areas of the organ of Corti. At the base of the gerbil cochlea^16,17^, for example, responses measured at sites near the outer-hair cell (OHC) and Deiters’ cell (DC) regions (Fig. 1A), are larger in amplitude and have broader frequency selectivity than at sites near the BM. Here I show that comparable discrepancies also occur in OoC impulse responses at the apex of the chinchilla cochlea.

The histological cross-section in Fig. 1A shows an OoC cross-section rotated to approximate the incidence angle of the optical axis on the B-scans (red dashed lines in Fig. 1B and C). Figure 1A depicts cellular components not usually discernible on B-scans (e.g., Fig. 1B and C) of apical cochlear regions, such as Hensen’s cells (HC) as well as OHCs and DCs.

Figure 1B and C display B-scans obtained at the apex of two chinchilla cochleae from the right ears. The x-axis corresponds to the field of view (FOV) and the y-axis to the depth inside the cochlea. Several structures can be visualized in Fig. 1B and C: scala vestibuli (SV), Reissner’s membrane (RM), scala media (SM), scala tympani (ST), and basilar membrane (BM). The otic capsule appears prominently in the B-scan. (At the center of the B-scan, FOV = 0.5 mm, the otic capsule is located between 0.6- and 0.9-mm depth.). The red dashed line indicates the optical axis in which measurements were obtained.

Responses to clicks at the apex of the chinchilla cochlea show differences across multiple OoC locations (FOV = 0.5 mm). The waterfall plot in Fig. 1D displays OoC responses to 70-dB pSPL clicks measured at eight equidistant locations at the depths indicated in the figure. (Each of the click responses in Fig. 1D represents an average of all the click responses recorded inside a 38-µm region.) Locations numbered 1, 2, and 3 in Fig. 1B correspond approximately to the HC, OHC/HC and BM regions, respectively (Fig. 1A). Notice that the response amplitudes are not the same across the recording sites but tend to decrease in the direction of the BM, which is located approximately at depth = 1.3 mm. Whereas the largest response was obtained at site 1, near the Hensen’s cell region, the smallest one was measured near the BM (site 3). Results in Fig. 1C and E originate from another cochlea and share similarities with those in Fig. 1B and D. Seven average responses to clicks are shown in the waterfall plot in Fig. 1E. The positions of four of those recordings (1–4) along the OoC are also indicated in Fig. 1C. Another important difference among the click responses as a function of depth in Fig. 1D and E is that the later part of the response (i.e., after 5 ms in Fig. 1D and after 10–12 ms in Fig. 1E) exhibit oscillations that are more prominent near the Hensen’s cell region (site 1) than near the BM (site 3). The reduced size of BM oscillations in this preparation can be due, in part, to the radial location of the recordings. Rhode and Recio (Fig. 9 in^18^) showed that vibration amplitudes at sites near the edges of the cochlear partition can be up to 20 dB smaller than those measured near the center. The fact that the angle between the optical axis of the measurement and the BM is approximately 45 degrees also contributes to the amplitude reduction.

### Spectral analysis of OoC responses to clicks

**Fig. 2:**
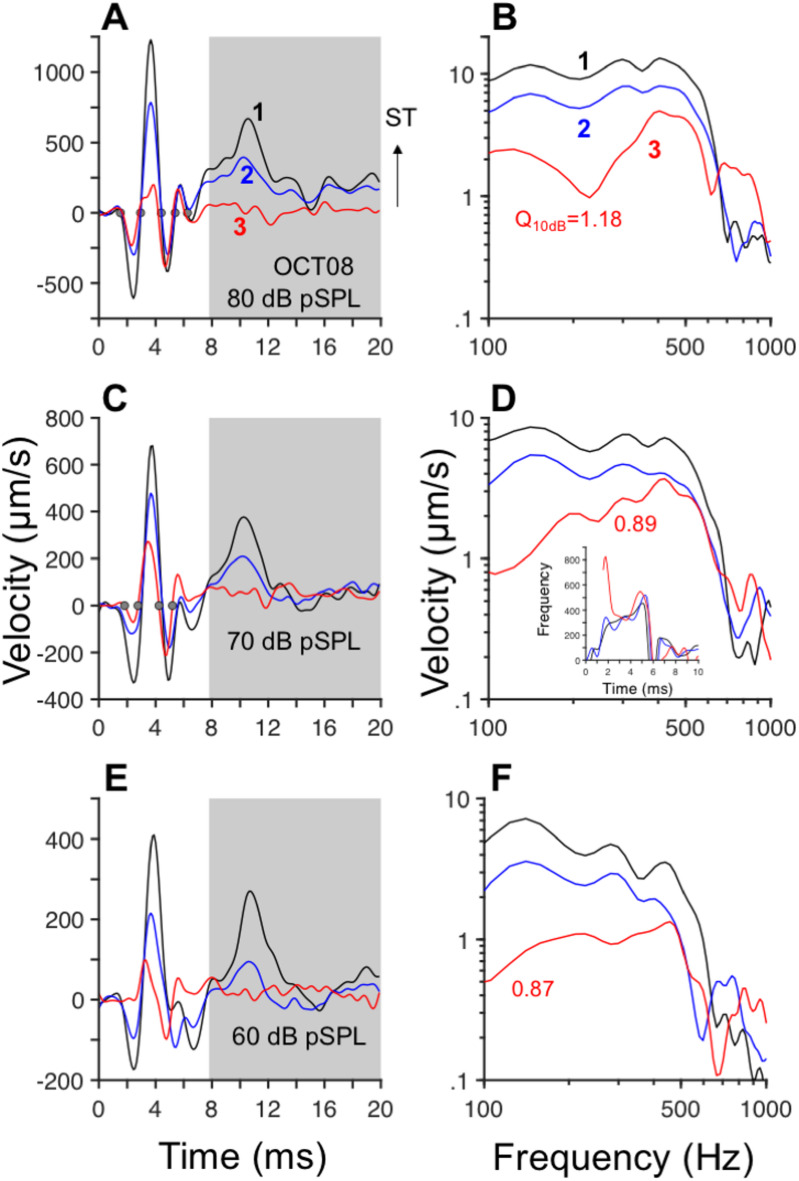
Differences in tuning across the organ of Corti (OoC) revealed by responses to clicks at different locations. Panels (**A**), (**C**), and E display time-domain responses to rarefaction clicks at three OoC locations (1: black lines; 2: blue lines; 3: red lines) at the stimulus intensities shown in each panel. Gray dots in A mark zero-crossing points of the waveform at location 1. Positive values in (**A**), (**C**), and (**D**) indicate motion in the direction of ST. Fourier transform magnitudes in (**B**), (**D**), and (**F**) were computed from the waveforms in (**A**), (**C**), and (**E**), respectively. Spectral analysis was performed with a 512-point fast Fourier transform (FFT). The inset panel in Fig. 2 displays instantaneous frequency -vs.-time for the waveforms in panel C using the same color conventions. Estimates of the quality factor, Q_10dB_, for site 3 appear on panels B, D, and F.

The left column in Fig. 2 displays average time-domain responses to rarefaction clicks recorded at sites 1, 2, and 3 in Fig. 1D at different click levels. The first negative oscillation in the responses in Fig. 2 was in the direction of scala media/scala vestibuli. Response waveforms recorded at sites 1 and 2 (black and blue lines, respectively, in Fig. 2) consist of two components: an initial segment, with band-pass tuning near CF, followed by a lower-frequency segment (shaded regions in Fig. 2A, C, and E). The amplitudes of the oscillations in the shaded regions are smaller near the BM than at more distant sites, regardless of stimulus level. Instantaneous frequency analysis performed using the five zero-crossing points shown in Fig. 2A (filled circles), for example, yields estimates of 348, 346, 504, and 576 Hz. That is, the instantaneous frequency of the initial component of the waveform increases as a function of time, until it reaches a value close to CF. By contrast, zero crossings in Fig. 2C (filled circles) indicate a decrease in frequency as a function of time for the waveform recorded at site 3. This observation is confirmed by the results of the instantaneous frequency analysis, using the Hilbert transform^3^, for the waveform recorded near the BM (site 3), as shown in the inset of Fig. 2D (red line). Whereas the instantaneous frequency of the BM motion in Fig. 2D starts at frequencies above CF and decreases toward CF (i.e., a downward glide), instantaneous frequencies of the responses recorded at sites 1 and 2 (black and blue lines, respectively, in the same inset panel) exhibit an upward glide.

Frequency selectivity of sound-evoked vibrations in the OoC was studied using the Fourier transform. Results of the Fourier analysis performed on the time-domain responses in Fig. 2A, C, and E are shown in Figs. B, D, and F, respectively, using the same color code. Irrespective of the stimulus intensity, Fourier transform amplitudes of the responses recorded at sites 1 and 2 (black and blue lines, respectively) exhibit a low-pass shape with a 400–500 Hz cutoff frequency. Responses recorded near the BM (red lines in Fig. 2B, D, and F), have band-pass tuning centered at 400–500 Hz. (Note that the band-pass shape of the BM velocity responses to clicks in Fig. 2 becomes low pass for BM displacement responses.) This difference in tuning has also been observed in BM responses to tones at the apex of the chinchilla^15^. The frequency selectivity of the responses near the BM (site 3) was quantified using the $Q$(quality) factor, i.e., the ratio of the center frequency of a band-pass filter to its bandwidth. (The higher the $Q$, the higher the frequency selectivity, or tuning, of the underlying filter.) ${Q}_{10dB}$ values shown in Fig. 2B, D and F increase with stimulus level.

**Fig. 3:**
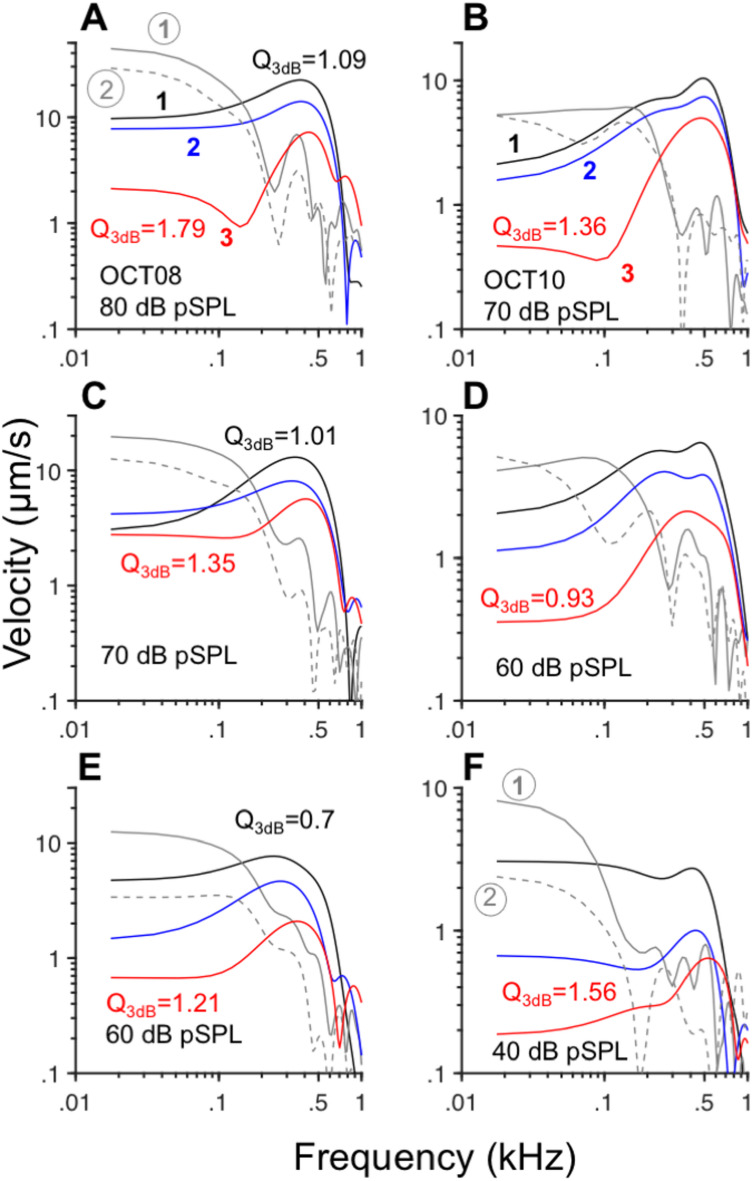
Spectro-temporal analysis of OoC responses to clicks reveals the presence of low- and band-pass components. Panels (A), (C), and E display Fourier transform amplitudes of the initial segment (from 0 ~ 8 ms) of the waveforms in Fig. 2. Colors indicate the recording sites (black lines: 1, near Hensen’s cell region; blue lines: 2; red lines:3, near the BM). Gray lines represent amplitudes of the late response components (from the shaded regions in Fig. 2) recorded at sites ① (continuous gray) and ② (dashed gray). Results in panels (B), (D), and (F) were obtained from click responses in a second cochlea, recorded at locations similar to those in (A), (C), and (E).

As described above, the impulse responses to clicks at the apex of the chinchilla cochlea can be separated into two distinct segments, corresponding to the unshaded and shaded portions of the waveforms (Fig. 2). The initial segment (preceding 8–10 ms in Fig. 2) consists of an oscillation with ringing characteristic of a band-pass filter. Response components later than 8–10 ms (in the shaded regions of Figs. 2) consist of a low-frequency component. Spectral analysis performed on the initial and final segments of the response waveforms in Fig. 2 are shown in Fig. 3A, C, and E. Fourier transform amplitudes of the initial components of the waveforms in Fig. 2A, C, and E display a band-pass shape centered at around 400–500 Hz (Fig. 3A, C, and E, respectively). Response amplitudes recorded near the Hensen’s cells and BM regions (black and red lines in Fig. 3A, C, and E, respectively) show an increase in frequency selectivity as a function of stimulus level, as indicated by the increase in Q_3dB_ values. (Due to the shape of the response amplitudes, it was not always possible to estimate Q_10dB_ coefficients, which are usually reported in the analysis of mechanical and neural responses of the auditory periphery.) The second component of the click responses, in the shaded region of Figs. 2, exhibits a low-pass shape with cutoff frequencies of around 100–200 Hz, as shown by the continuous and dashed gray lines in Fig. 3A, C, and E, which correspond to the recordings from sites 1 and 2, respectively.

Fourier transform amplitudes of the initial and final segments of the responses from a different cochlea also exhibit band-pass and low-pass profiles, respectively (right column in Fig. 3). Fourier analyses of the initial segments recorded near the Hensen’s cells region (black lines in Fig. 3B, D, and F) indicate the presence of a band-pass filter at 70 dB SPL. At lower stimulus levels, the frequency selectivity of the filter decreases until it becomes low pass at 40 dB SPL (Fig. 3F). Filtering near the BM (red lines in Fig. 3B, D, and F) is band-pass, regardless of stimulus level. The increase in frequency selectivity with stimulus level of the BM, however, is nonmonotonic as determined by the values of Q_3dB_. Gray lines in Fig. 3B, D, and F indicate the low-pass profile of the second component of the responses to clicks (with a cutoff frequency of around 100–200 Hz).

**Fig. 4:**
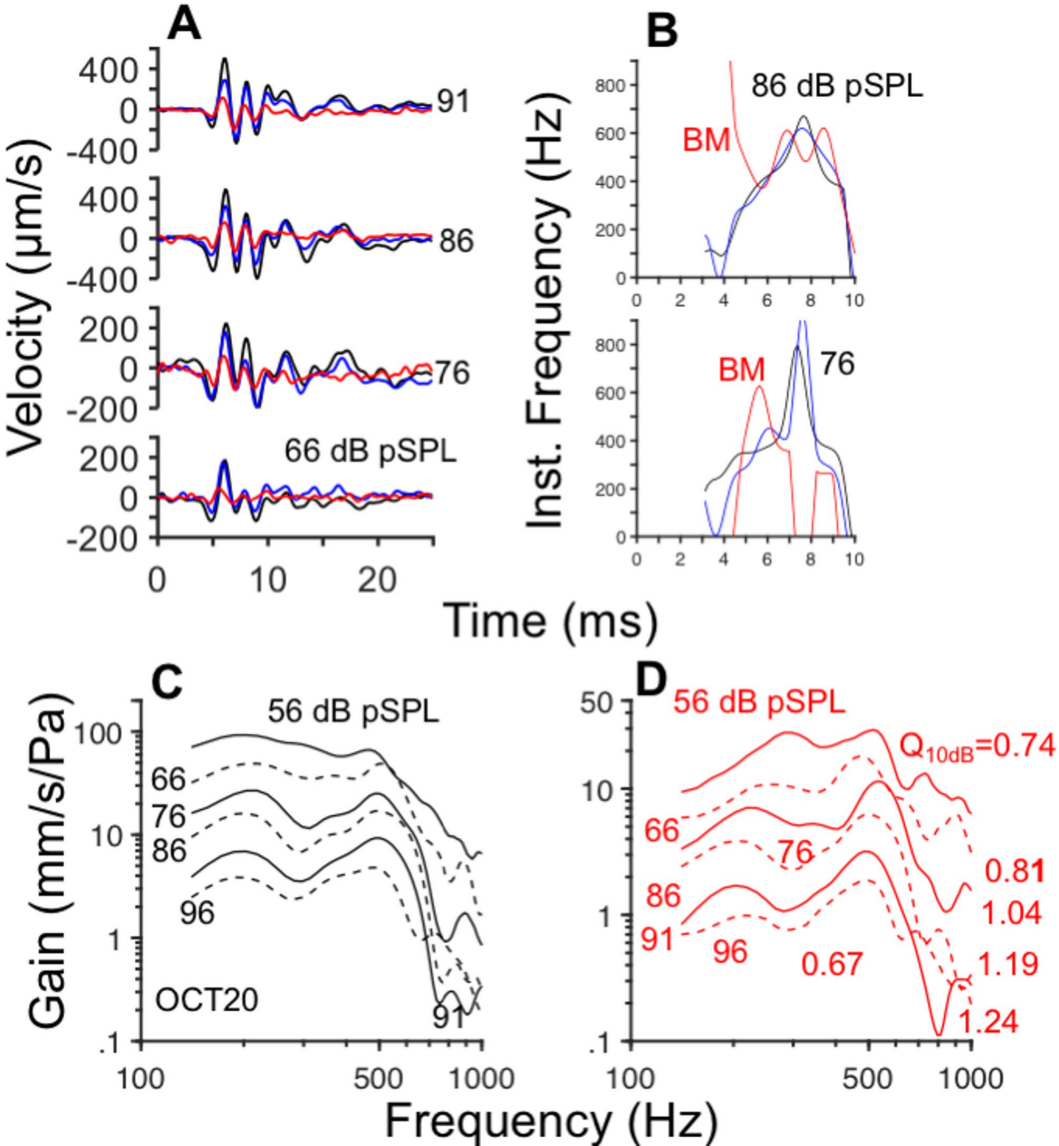
Changes in cochlear gain with stimulus level. Panel (**A**) displays OoC time-domain responses to rarefaction clicks at the intensity levels indicated next to each waveform. The color convention (black, blue, and red is the same as in Fig. 2). Instantaneous frequency-vs.-time plots at two intensity levels are shown in (**B**). Panels (**C**) and (**D**) display iso-intensity curves normalized to the stimulus level (in Pascal) recorded at sites 1 and 3, respectively. Results in panel (**D**) were obtained after averaging the responses recorded in sites 3 and 4 (see Figs. 1C and E). Q_10dB_ estimates appear on panels C and D.

Figure 4A displays OoC click responses from cochlea OCT20 at several intensity levels. Waveforms in Fig. 4A consist of two components, just as the results in Fig. 2. Results of the instantaneous frequency analysis of two sets of response waveforms, obtained at 86 and 76 dB pSPL, are shown in the two panels of Fig. 4B. In both plots, downward glides were found for the responses recorded near the BM (red lines in Fig. 4B), but not for the waveforms measured in sites 1 and 3. Figure 4C and D display Fourier transform amplitudes from sites 1 and 3 (black and red lines in Fig. 4A) normalized to the click stimulus intensity (in Pascals). Gain-vs.-frequency curves in Fig. 4C and D do not overlap each other: gains decrease in value with increases in stimulus level. Gain functions near the Hensen’s cells (site 1) have a predominantly low-pass shape with a cutoff frequency of around 500 Hz (Fig. 4A and B) whereas gain functions near the BM exhibit a band-pass profile (Fig. 4C and D).

Quality factors, ${Q}_{10dB}$, shown in Fig. 4D increase with stimulus level, except for the last stimulus level (96 dB SPL). Similar increases in ${Q}_{10dB}$ values with stimulus level were found in the results of Fig. 2. The increase in frequency selectivity with stimulus level at the apex of the cochlea contrasts with the decreases in frequency selectivity with stimulus level at the base of the chinchilla cochlea^3,4,18,19^.

**Fig. 5:**
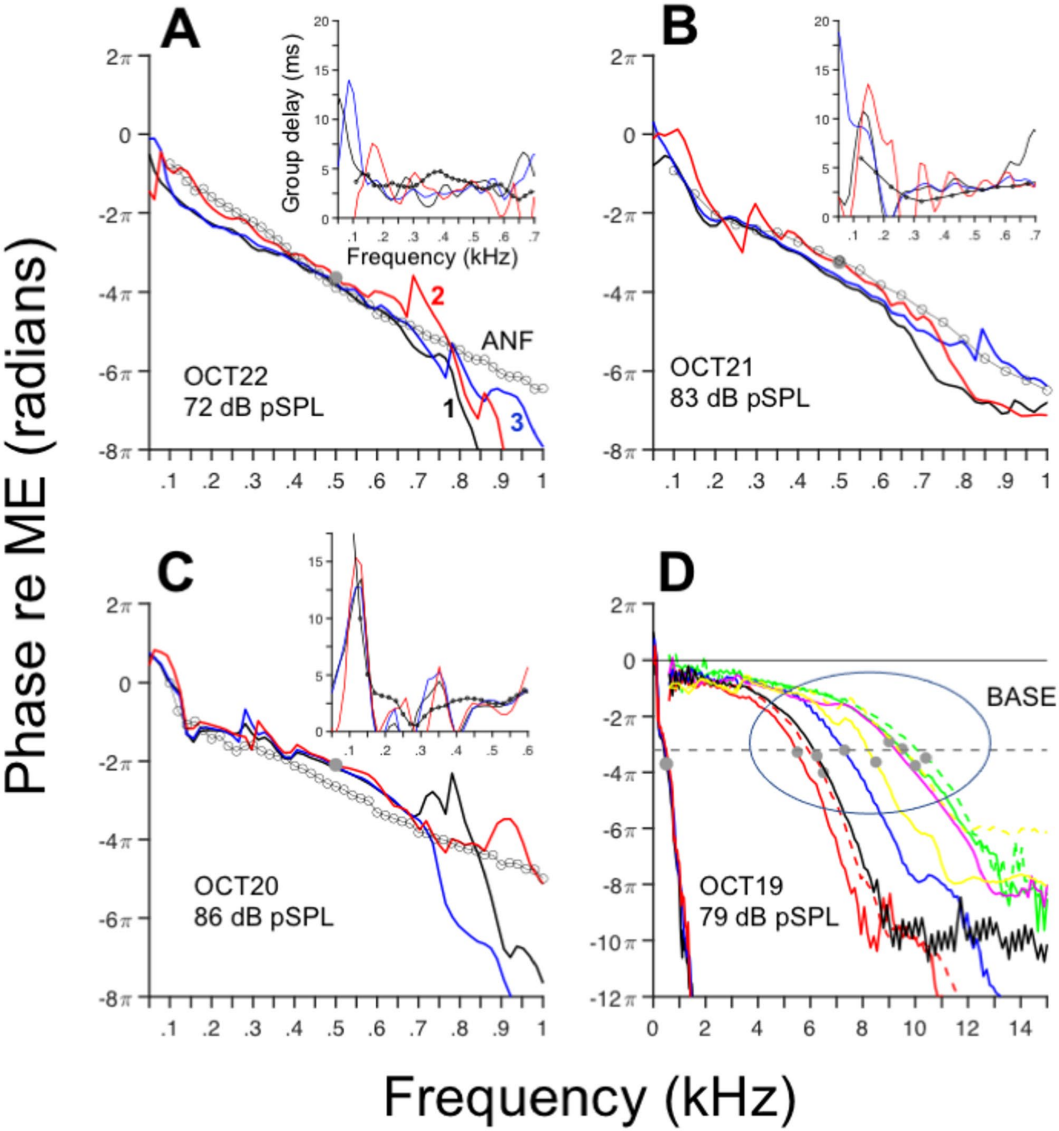
Response phases relative to middle-ear motion exhibit an inflection point around 150–200 Hz. Continuous lines represent OoC response phases relative to middle-ear motion for the cochleae indicated in each panel. Phase leads and lags are represented as positive and negative values, respectively. Black, blue and red lines represent phases measured around the HC, OHC/DC and BM regions, respectively. Continuous lines with circles in A-C represent ANF response phases relative to rarefaction corrected for a 1-ms synaptic/neural delay. (**A**): N1405, u19, CF = 366 Hz, 70 dB SPL, (**B**): N14097, u20, CF = 496 Hz, 70 dB SPL; (**C**): N1407, u18, CF = 553 Hz, 40 dB SPL) Insets display group delays as a function of frequency. Panel (**D**) displays phase-vs.-frequency curves measured at the apex (OCT19) alongside BM phase responses to clicks at the base (1^st^ turn) of other chinchilla cochleae. Filled circles indicate CF.

Response phases to clicks, obtained using Fourier transforms and expressed relative to middle-ear (ME) motion, exhibit increasing phase lags as a function of frequency (Fig. 5) regardless of the OoC region. In general, response phases as a function of frequency display a concave (“U-shaped”) pattern with an inflection point at around 150–200 Hz. Phases obtained from auditory nerve fiber (ANF) responses to tones (continuous lines with open circles in Fig. 5A-C) and response phases to clicks in Fig. 5 share similarities in their shapes and values. In fact, the inflection points at 150–200 Hz observed in the mechanical vibrations also occur in the ANF responses. At near-CF frequencies (400–500 Hz), group delays obtained from OoC vibratory responses to clicks and from ANF responses to tones have comparable values (around 2.5 ms), as shown in the inset panels in Fig. 5A-C. Moreover, group delays obtained from ANF responses to tones and vibratory responses to clicks exhibit an increase in delays at frequencies below 100–200 Hz (insets in Fig. 5A-C).

Figure 5D illustrates a phase-vs.-frequency curve obtained from click responses at the apex of chinchilla (OCT19) as well as phase curves extracted from click responses measured at the first turn of several chinchilla cochleae^4^. The overall shapes of the phase-vs.-frequency curves at the base of the cochlea differ from those at the apex. However, phase lags at CF, namely around $3\pi$ radians (1.5 cycles), are approximately the same (filled circles in Fig. 5).

Phases-versus-frequency curves obtained from the basilar membrane (BM, red lines in Fig. 5; see also^20^) lead the HC and OHC response phases (black and blue lines, respectively). One discrepancy between our findings and those in^20^ lies in the relationship between BM and OHC phases. While BM phases also lead OHC phases in this study, they were reported to lag OHC phases in^20^.

### Effects of death on OoC responses to clicks

**Fig. 6:**
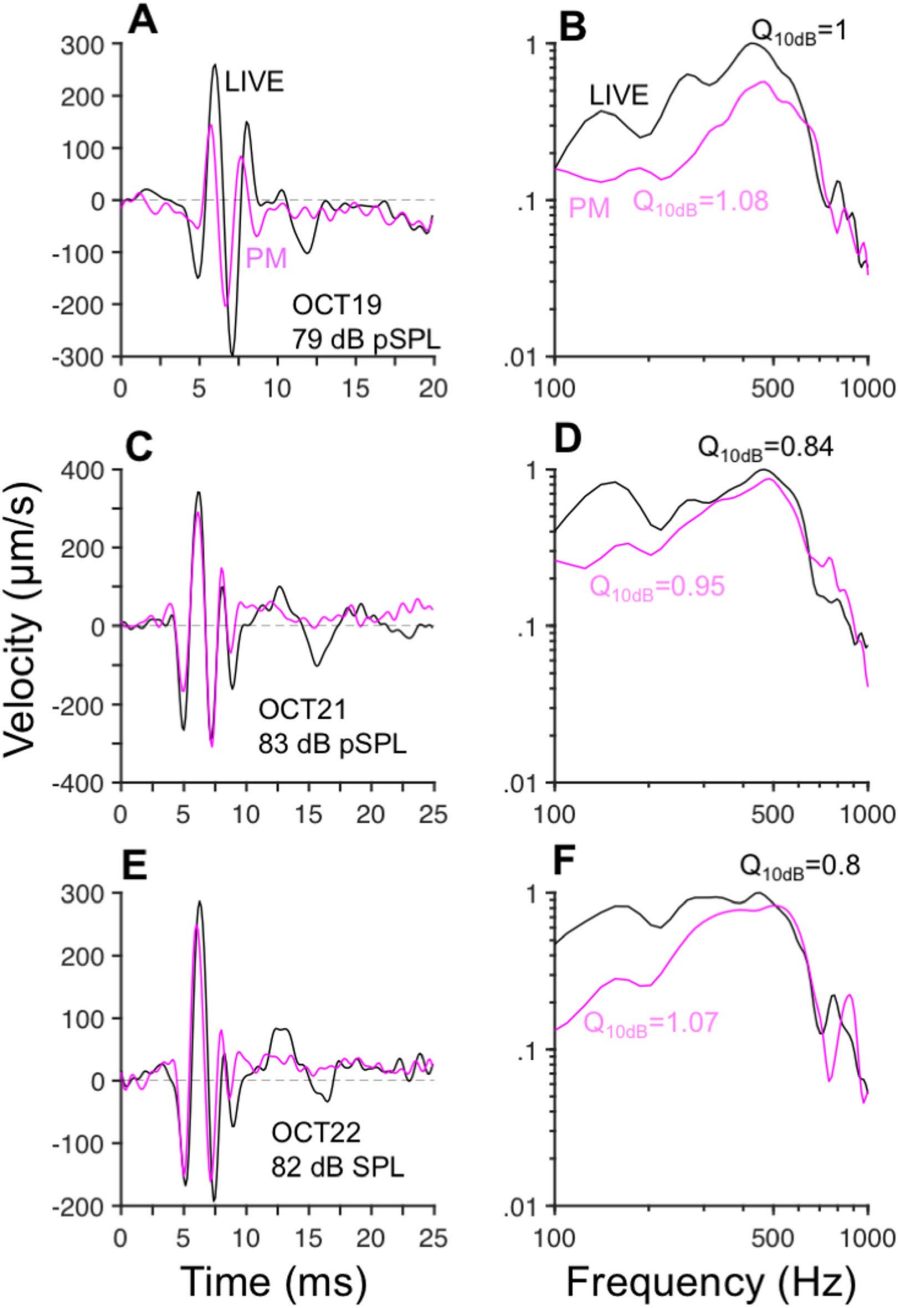
Postmortem effects on OoC responses to clicks. Panels (**A**), (**C**), and E display time-domain recordings near the Hensen’s cells region, measured before (LIVE: black lines) and after death (PM: magenta lines). Normalized Fourier transform amplitudes in (**B**), (**D**), and (**F**) were computed from the waveforms in (**A**), (**C**), and (**E**), respectively.

The effect of death on OoC responses to clicks was studied in several preparations. Figure 6A, C, and E display OoC responses to clicks recorded at site 1, located near the Hensen’s cells region, in three cochleae before (black lines) and after (magenta lines) death of the animal. The main effect of death occurs in the later part of the response (after 10 ms), where low-frequency oscillations are nearly abolished. The effect on the early response component is variable: whereas in one cochlea (Fig. 6A and B) the postmortem (PM) response was smaller than the response of the live preparation, in the other two preparations (Figs. 6C-F) the initial segment of the response (before 10 ms) remained nearly constant.

Fourier transform amplitudes of the responses in Fig. 6A, C and E are shown in Fig. 6B, D, and F, respectively. In all cases, the CF of the responses remains nearly the same after death. Postmortem, the response magnitudes lower than CF decrease systematically with decreasing frequency, resulting in an increase in frequency selectivity, as indicated by the increase in Q_10dB_ factors in the PM recordings.

### Travel times of OoC responses to clicks

**Fig. 7:**
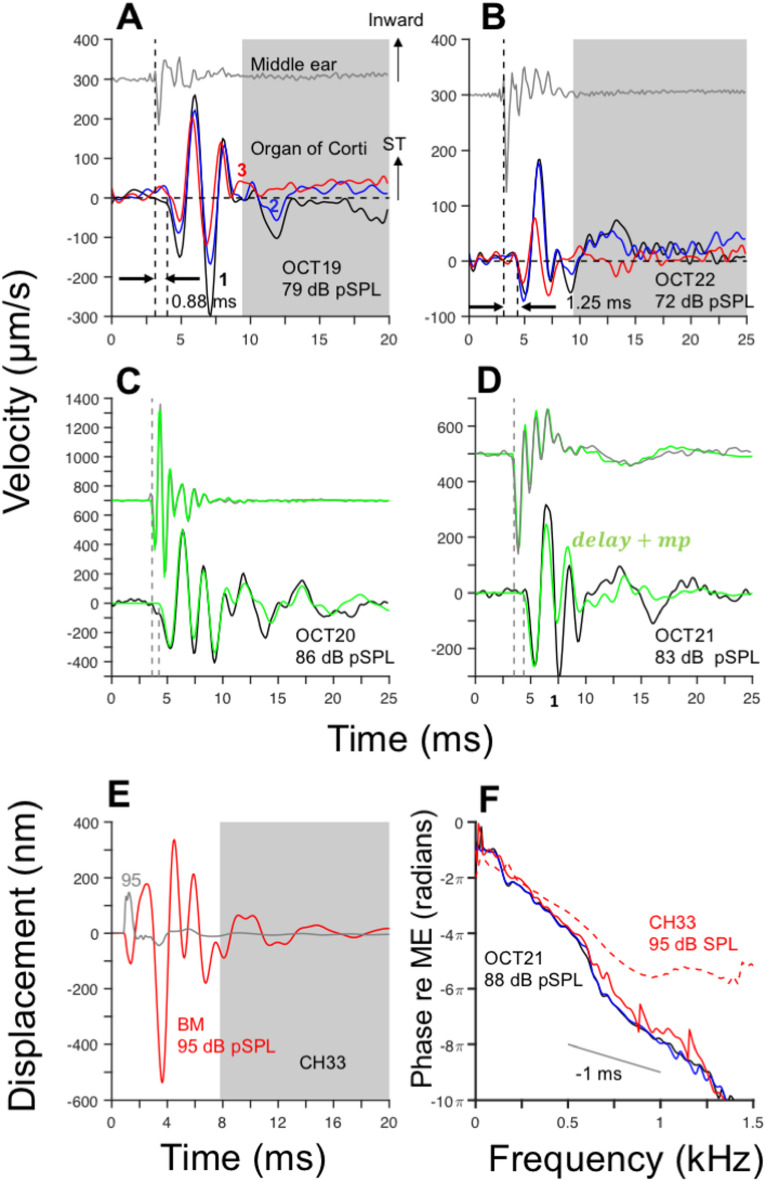
OoC responses to rarefaction clicks reveal a slow traveling wave. Top traces (gray lines) in (**A**) and (**B**) represent middle-ear (ME) time-domain responses to rarefaction clicks. Lower traces in (**A**) and (**B**) show OoC responses at three locations (1: black; 2: blue, 3: red). Negative ME responses indicate motion away from the cochlea. (Middle-ear responses in panels (**A**-**D**) were shifted by an arbitrary amount for clarity.) Negative OoC responses indicate motion towards scala vestibuli/scala media. Response onsets are indicated by vertical dashed lines. Top and lower curves in (**C**) and (**D**) display ME and OoC responses (around HC regions), respectively. Superimposed green lines represent signal-front delay plus minimum-phase approximations ($delay+mp$) of each response. BM and umbo responses to clicks, measured after a cochleostomy, are shown using red and gray lines, respectively, in panel (**E**). Panel F displays OoC (from chinchilla OCT21) and BM (from chinchilla CH33 from ^5^) response phases relative to ME motion. The gray line indicates a slope of -1 ms.

OoC responses to clicks recorded at the apex of live animals do not start immediately upon the first oscillation of the ME ossicles (Fig. 7). Rather, as expected from the traveling wave theory formulated by Georg von Békésy^21^, they are delayed relative to the onsets of ME vibration. Onset times have been previously defined^3,4^ as the time at which the initial oscillation of the vibration response reaches 20% of the maximum absolute value. Vertical dashed lines in Fig. 7A and B indicate the onset times at which the initial oscillation reaches 10% of the minimum value of the initial negative peak. The difference between the OoC and middle-ear response onsets equals the travel time^21^: 0.8 and 1.25 ms for the results in Fig. 7A and B, respectively. The average travel time for the cohort (N = 6) was 1.12 ms. Travel times are independent of the region inside the OoC (1: Hensen’s cells, black lines; 2: OHC/DC, blue lines; 3: BM, red lines). Travel times, as defined by Békésy, are usually referred to as signal-front delays in the signal processing literature^22,23^. Signal-front delay is independent of frequency and, in the time domain, represents “the delay of the beginning, or *front*, of a signal”^22^.

The determination of the response onsets in Fig. 7A and B is based on an arbitrary “10% criterion” and should therefore be considered as approximation to the true values, Measuring the true signal-front delay is hampered by the noise level in the click response. To address this, signal-front delays were also estimated by obtaining a minimum-phase approximation of the cochlear impulse response (see Methods sections). Cochlear impulse responses were modeled by a frequency-independent delay followed by the minimum-phase impulse response. The length of the delay corresponds to the duration of the travel time, that is, the signal-front delay.

Figure 7C and D show middle-ear (gray lines) and OoC click responses (black lines), along with signal-front delay plus minimum-phase approximations (green lines: *delay* + *mp*) to the responses. Signal-front delays measured from the results in Fig. 7C (OCT21) and D (OCT20) were 0.88 ms and 0.62 ms, respectively. ME click responses were well fit by a *delay* + *mp* model and were consistent with previous results^14^. The pure delay of the ME responses in this case corresponds to equipment delay. Overall, OoC click responses were reasonably approximated by a delay plus a minimum-phase filter, particularly during the initial response oscillations.

BM responses to clicks, recorded after a cochleostomy in the third turn of the chinchilla cochlea^5^, were also analyzed. Figure 7E shows umbo (gray line) and BM responses (red line) to condensation clicks at 95 dB pSPL, respectively. (ME responses shown in^5^ were measured for 75 dB SPL clicks. The waveform in Fig. 7E was linearly extrapolated to 95 dB SPL.) BM responses to clicks consist of two segments, similar to the OoC responses to clicks presented in this work. The delay between BM and umbo response onsets is approximately 50 µs, a value comparable to the travel time of sound waves in water, as noted by the authors in^5^.

Figure 7F compares BM responses phases relative to middle-ear motion (red dashed lines) with phases computed from OoC responses to clicks in OCT21. BM response phases from chinchilla CH33 exhibit a phase plateau starting at frequencies around 800 Hz—a feature not observed in OoC response phases. When comparing responses phase recorded in one guinea pig cochlea before and after sealing the cochlea, the authors found a similar pattern in the phase-vs.-frequency functions under both experimental conditions [Fig. 7 in^5^]. The phase plateau in the high-frequency region is attributed to the fast-traveling wave as mentioned in^5^.

Despite the presence of a fast-traveling wave in their recordings, Cooper and Rhode^5^ demonstrated important nonlinear properties of BM response to clicks at the apex. Fourier analysis of the responses (not shown here) revealed a CF of approximately 660 Hz, with a frequency selectivity increasing as the click level decreased. Moreover, instantaneous frequency analysis of the click responses revealed upward glides. This behavior contrasts with the findings in this report and in^15^.

**Fig. 8:**
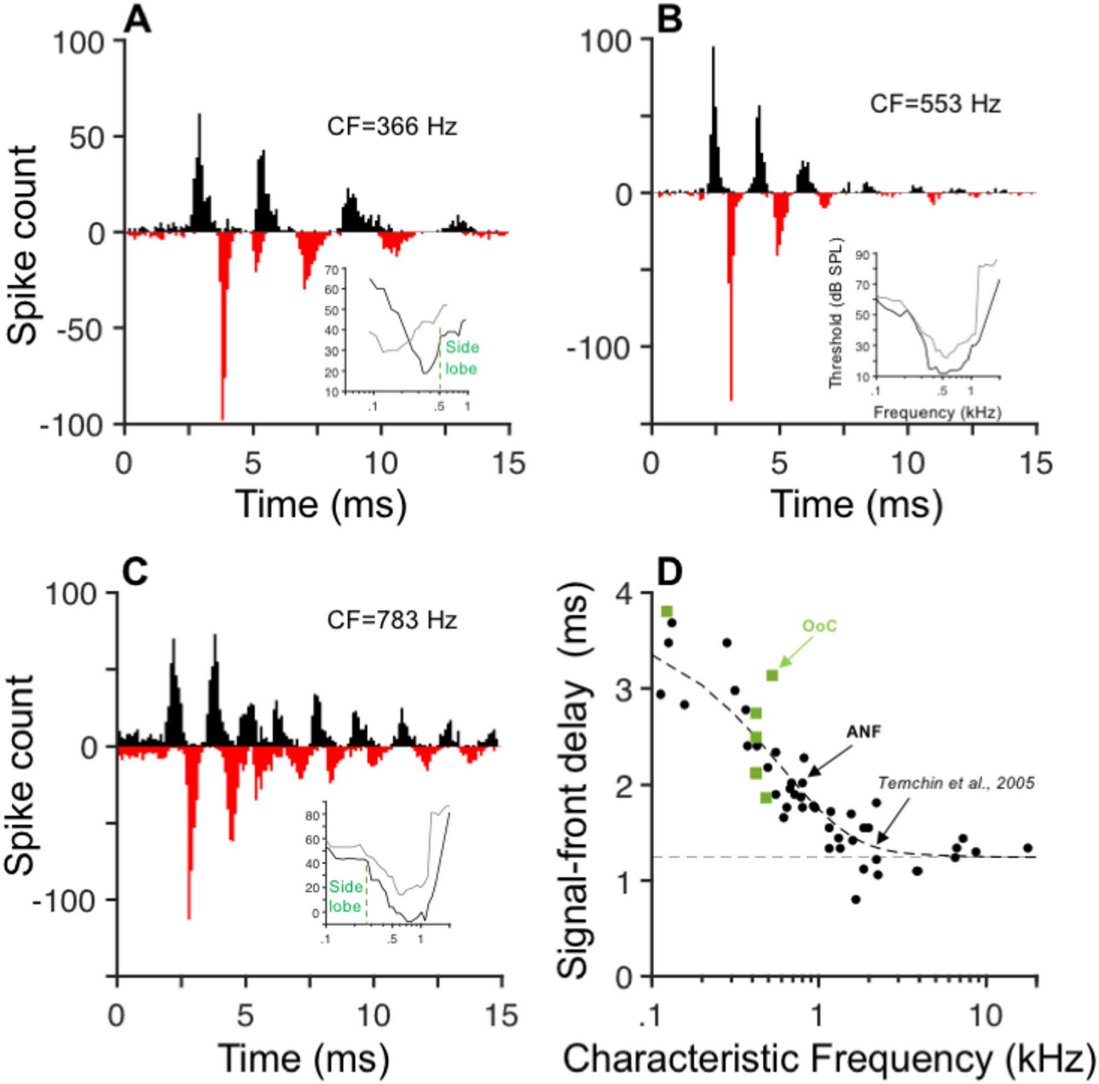
Signal-front delays obtained from auditory nerve fiber (ANF) and OoC click responses are similar. Panels (**A**), (**B**), and (**C**) display compound post-stimulus time histograms obtained from responses to rarefaction and condensation clicks of three ANFs. Insets show tuning curves: black lines correspond to the ANF shown in each panel; gray lines represent tuning curves of other ANFs with similar CF. Signal-front delays estimated from ANF responses to rarefactions clicks are shown in panel D (filled circles), along with a fit to ANF responses to clicks (dashed line): $\tau \left(ms\right)=1.44+2.402{e}^{-1.699CF}+0.083{e}^{-0.269CF}.$ The constant term of 1.44 reflects the average synaptic and propagation delays plus hardware delays (indicate by the dashed horizontal line in D).Signal-front delays of OoC responses to clicks are shown in (**D**) as filled green rectangles after adding 1.44 ms to their values.

Compound histograms^24^ computed from the responses of three ANFs to rarefaction (black) and condensation (red) clicks are shown in Fig. 8A-C along with their respective tuning curves in the insets (black lines). Gray lines in the inset panels display tuning curves from different ANFs with similar CFs. Although the ANFs in Fig. 8A-C arise from sites in the third turn of the chinchilla cochlea, that is, the “apex”, there are clear differences in their shapes. Tuning curves in general display asymmetries in their shapes around CF, with “side lobes”^25^ either above CF (e.g., inset in Fig. 8A) or below CF (e.g., inset in Fig. 8C).

Onset delays of ANF responses to clicks were defined at the time at which the spike count reached 20% of the first histogram peak. Signal-front delays of a sample of ANFs^26^ are plotted as a function of CF together with OoC delays shifted by + 1.44 ms (black circles and green squared, respectively, in Fig. 8D). The value added to OoC delays (dashed horizontal line in Fig. 8B) corresponds to average synaptic and propagation delays plus hardware delays in the instrumentation system^26^. A fit to the delays of ANF responses to clicks from a larger sample^27^ is also shown in Fig. 8D.

### OoC responses to frequency-modulated and white noise stimuli

**Fig. 9:**
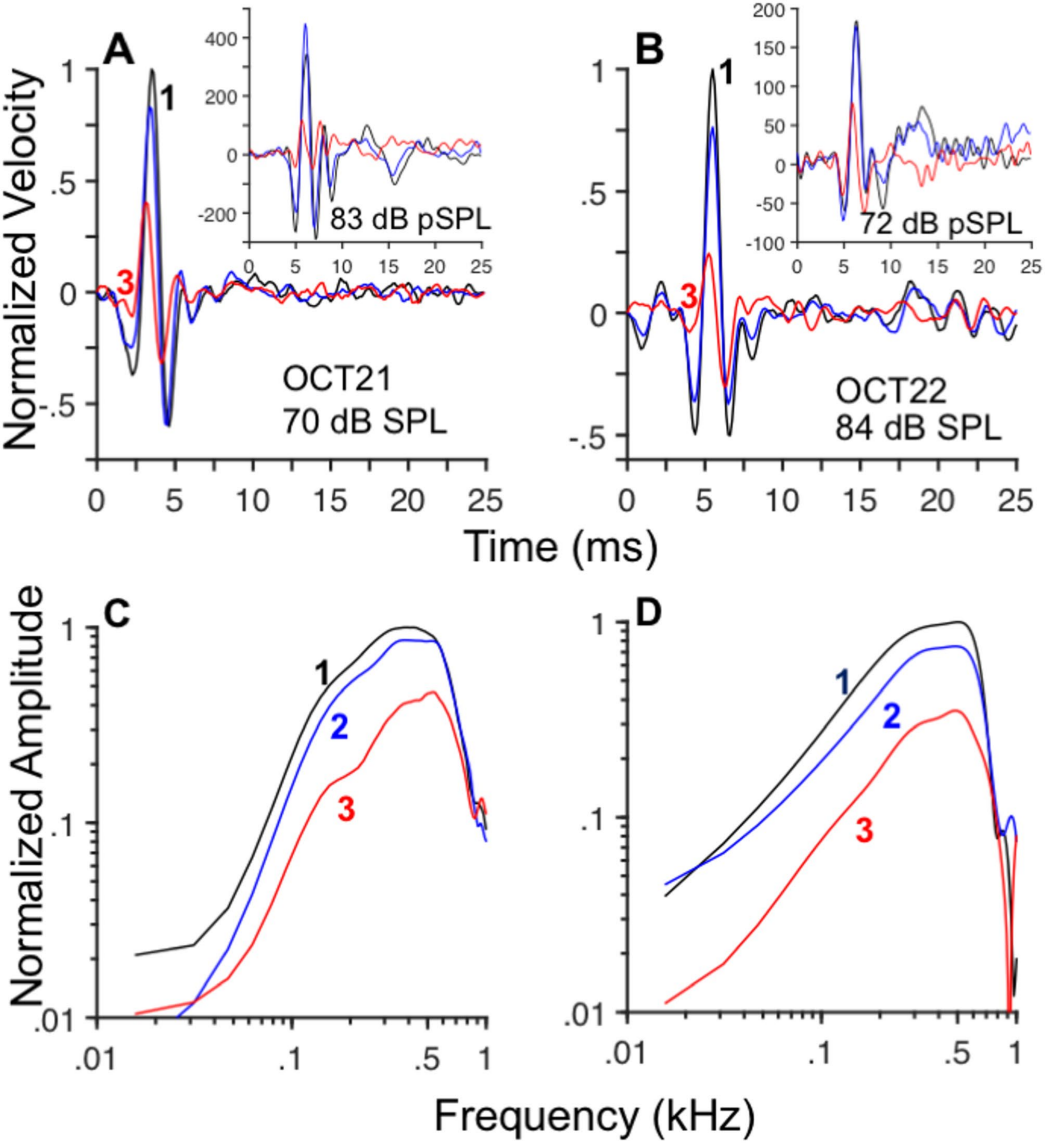
OoC responses to clicks, exponential-swept tones, and white noise at the apex of the chinchilla cochlea. Panels (**A**) and (**B**) display indirect impulse responses obtained from responses to exponential-swept tones and white noise, respectively. Insets in both panels show click responses recorded from the corresponding cochlea. Panels (**C**) and (**D**) exhibit normalized Fourier transform amplitudes of the responses shown in panels (**A**) and (**B**), respectively. Numbers in C and D indicate the OoC region where each recording was made (1: Hensen’s cells region; 2: OHC/DC region; 3: BM region).

Indirect impulse responses of the OoC were obtained using exponential-swept tones and broadband noise as stimuli (Fig. 9A and B, respectively). In general, the shapes of both types of indirect impulse responses are similar: they exhibit waveforms characteristic of band-pass systems and lack the low-frequency component that is a feature of click responses (insets in Fig. 9A and B).

Fourier transform amplitudes of the responses in Fig. 9A and B are shown in Fig. 9C and D, respectively. For all three OoC regions (1, 2 and 3), the Fourier analyses show band-pass tuning centered at around 500 Hz, in contrast to the low-pass profile of OoC responses to clicks (Figs. 2 and 3).

For a linear system, the first-order Wiener kernel is identical to the system’s impulse response. Given the nonlinear properties of the cochlea, the differences between the kernels and the click responses in Fig. 9 are not surprising but warrant some explanation. The first-order Wiener kernel of a nonlinear system is a component of the impulse response and contains the linear part of the response as well as contributions from higher odd-order nonlinearities^28^.

## Discussion

The impulse response of a linear system completely characterizes its input–output properties so that its Fourier transform is identical to the transfer function of that system, usually obtained using single tones. For nonlinear systems, responses to tones and the spectra of responses to clicks are not necessarily equivalent. At the base of the chinchilla cochlea, for example, impulse responses include spectral notches near CF that are not found using single tones^4^. Thus, for nonlinear systems impulse responses complement information provided by responses to single tones. Moreover, impulse responses exhibit time-domain representations of important cochlear attributes such as signal-front delays as well as time-varying dynamics, which are less apparent in responses to single tones. Signal-front delays are an essential component of the traveling wave theory proposed by Békésy, yet their determination has been previously marred by the interaction between fast and slow traveling waves due to experimental conditions, such as cochleostomies^5^. Nevertheless, analysis of the click evoked responses in^5^ reveal important nonlinear properties of click-evoked responses at the apex of the chinchilla cochlea.

The OoC responses to clicks measured in the current study originate from a site in the third turn of the chinchilla cochlea, corresponding to a CF in the range of 400–500 Hz. In contrast, the BM responses to clicks reported by Cooper and Rhode^5^ arise from a nearby site in the third turn but with a higher CF (~ 660 Hz) and exhibit different nonlinear properties. The findings of Cooper and Rhode, along with those presented here, suggest that these two locations along the tonotopical map of the cochlea exhibit distinct sound processing characteristics.

Furthermore, Rhode and Cooper reported responses to single tones at a location with a CF of 400 Hz in the chinchilla [Fig. 11B in 29], which showed changes in frequency selectivity with stimulus level similar to those observed here and in^15^. Discrepancies in changes in frequency selectivity with stimulus level have also been observed among recordings in the second and third turns of the guinea pig cochlea^8,15^, at sites with CFs below 700 Hz. In other words, nonlinear processing of sounds differs within the apex of the cochlea.

Unpublished observations of responses to clicks at several locations in the first turn of the chinchilla cochlea^4^ also indicate differences in responses across various sites. Taken together, these observations suggest the existence of a tonotopic continuum from base to apex. In other terms, “there are not abrupt transitions,” as noted in^30^ when discussing anatomical differences across the chinchilla cochlea.

Mechanical responses to clicks at the base and at the site of the chinchilla cochlea where the recordings in this work were performed differ in at least three respects. First, due to the presence of the traveling wave, the latencies of the responses increase from a few microseconds at the base to at least 1.1 ms at the apex. Second, upon death the response bandwidth increases at the base but decreases at the apex. Third, frequency tuning increases with increasing stimulus level at the apex but not at the base. Whether these last two properties hold for other locations in the third turn of the cochlea remains to be proven.

The present study is the first to describe responses to clicks at the apex of intact cochleae in a laboratory species with low-frequency hearing comparable to that of humans. The average travel time to the site with a 500-Hz CF, estimated here in six cochleae, is approximately 1.1 ms. This estimate, which was obtained using an arbitrary criterion for the determination of the response onset, was accurately predicted by the latency of ANF responses to rarefaction clicks in the chinchilla (1.1 ms at CF = 500 Hz; Fig. 10B in^27^) after compensation by a 1.244 ms neural delay.

The average latency estimated here is also similar to the 1-ms delay reported by Békésy at a site of the human cochlea with a “resonant” frequency of 500 Hz (Fig. 11 in^21^). However, the distances from the stapes to the recording sites in the human and chinchilla cochleae are 25 and 15 mm, respectively.

The representation of the cochlear click responses using minimum-phase filters provides also estimates of signal-front delays (Fig. 7C and D). The average signal-front delay computed using this method equals 1.17 ms, which is remarkably similar to the estimate indicated above. Moreover, results of this analysis indicate that the filtering of sounds performed at the apex of the cochlea can be approximated by a signal-front delay followed by a minimum-phase filter.

The present report is also the first in which “fast” responses were not observed in live animals. Previous recordings of mechanical responses to clicks at the apex of the chinchilla cochlea^5^ exhibited very short latency onsets, in the order of 50 microseconds, apparently reflecting fast pressure waves (i.e., acoustic waves) propagating in the cochlear fluids (Fig. 7). These were interpreted (correctly, in retrospect) as an artifact of the cochlea being unsealed.

The shape of the phase curves (Fig. 5) is reminiscent of cochlear microphonic (CM) phases in the same species^31^. In both cases (OoC vibrations and CM), phase-frequency curves have two segments, with an inflection point at frequencies between 100 and 200 Hz: a low-frequency segment with high group delay and a high-frequency segment with lower group delay. By contrast, phase-vs.-frequency curves obtained from chinchilla ANF responses to single tones have inflection points above CF (Fig. 8 in^32^). Some of the phase curves in^32^, particularly those with CF > 1 kHz, have changes in curvature at frequencies around 100–200 Hz, similar to the results in Fig. 5. The shapes of the chinchilla CM phase functions, which Dallos^31^ showed differed from those in other laboratory species, was thought to result from the relatively large size of the helicotrema in the chinchilla.

Albeit unremarked by its authors, a report on vibrations at the apex of the chinchilla cochlea exceptionally illustrated an increase in frequency selectivity with stimulus level (Fig. 11B of^29^). It was shown recently that this is the normal behavior for responses to tones at locations with CFs in the 400–500 Hz range in the chinchilla cochlea and in the extreme apex of the guinea pig cochlea^15^. The present results for responses to clicks extend those findings and confirm that vibrations at certain sites of the third turn of the cochlea sharply deviate from what is known about BM filtering at the base of the chinchilla cochlea.

Postmortem effects on OoC responses to clicks at the chinchilla apex (Fig. 6), similar to those previously found in guinea pig (Fig. 2 in^8^), differ from those at the base of the chinchilla cochlea. Changes of BM responses at the base include a half-octave decrease in CF (contrasting with no CF change at the apex) as well as a decrease in frequency selectivity (contrasting with an increase in frequency selectivity at the apex).

Frequency selectivity in a positive-feedback model of apical processing of sounds also increases with stimulus level (Fig. 5 in^33^). The extent of the nonlinearity in that model, however, does not include frequencies around CF. In contrast, Khanna and Hao^34^ and Zinn et al^35^ proposed a negative-feedback model to explain the results of their experiments at the apex of the guinea pig cochlea, which show primarily linear growth with stimulus level across all stimulus frequencies. Their finding disagrees with the results in ^8^ obtained in intact guinea pig cochleae, in which nonlinear effects also occur at frequencies around CF. Nevertheless, whether the filtering process at the apex of the cochlea is based on a positive- or a negative-feedback mechanism requires further study.

Indirect impulse responses obtained using swept-tone and noise stimuli (Fig. 9) consist of a single component, with band-pass selectivity centered at the CF. Charaziak and Altoè ^36^obtained indirect impulse responses using frequency sweeps at cochlear regions with CFs around 8–9 kHz in mice. The frequency spectra of those indirect impulse responses resemble the frequency spectra of BM responses to clicks at the base of the chinchilla cochlea^3,4^.

Whereas first-order Wiener kernels (i.e., cross-correlation functions between a noise stimulus and a system’s response) consist of odd-order nonlinearities^13,28^, click responses also contain even-order nonlinearities. Given the similarities between the first-order Wiener kernels and the initial segment of OoC click responses (Fig. 9), it is likely that the second component that appears in many OoC responses to clicks (e.g., shaded regions in Figs. 2 and 7) is a manifestation of a quadratic nonlinearity. Rhode and Cooper^29^ reported baseline position shifts (i.e., DC shifts), which are, by definition, a manifestation of an even-order nonlinearity, in TM responses to short tones. More recently, Dewey et al^37^ observed similar DC shifts in responses to tones measured at both the TM and OHC-DC junction. Their Fourier analysis revealed strong second harmonic (2xfrequency) components, consistent with an even-order nonlinear mechanism. This suggests that the later part of the responses to clicks measured at or near the Hensen’s cells and OHC regions (e.g., shaded regions in Fig. 2A, C, and E) likely represent a time-domain representation of an even-order nonlinearity.

## Conclusions

The experimental results presented here are based on recordings from the third turn of the chinchilla cochlea, with an approximate characteristic frequency (CF) of 500 Hz. Frequency selectivity increases with stimulus level, consistent with changes in tuning observed in responses to tones at the same location.

OoC responses to clicks exhibit a high-frequency oscillation, tuned to approximately 500 Hz, followed by a low-frequency component. In postmortem recordings, the low-frequency component decreases substantially, contributing to an apparent increase in frequency selectivity. This apparent sharpening arises from the loss of the low-frequency component rather than from an improvement in the active processes of the cochlea.

First-order Wiener kernels obtained from responses to noise in the live animal do not show this low-frequency component. Since first-order Wiener kernels include linear components and odd-order nonlinearities, it is possible that the low-frequency component observed in the click responses is a manifestation of an even-order nonlinearity.

## Methods

### Animal preparation

Chinchillas obtained from a vendor in Tarragona, Spain, were anesthetized with ketamine (40 mg/Kg, IM) and xylazine (2 mg/kg, IM) with additional smaller doses applied as needed. Body temperature was kept at 38ºC by means of a heating pad under the control of a servomechanism. Tracheotomy and tracheal intubation were performed routinely but forced ventilation was seldom used. After the right pinna was resected, four screws were implanted in the skull and cemented to provide a stable fixation of the skull to a head holder. The right bulla was opened widely through a ventral approach to visualize the apical region of the cochlea. The tensor tympany was cut and the stapedius muscle was detached from its anchoring. In some animals, an opening of the bony ear canal over the tympanic membrane was made. The opening was sealed with a glass cover thus allowing a view of the umbo.

ANF responses to clicks were measured in a separate group of chinchillas (N = 5). Results from a previous analysis of this data set have been published in conference proceedings^26^, where the surgical methodology is described in detail. Briefly, chinchillas were initially anesthetized with Ketamine (100 mg/Kg, s.c.) and Dial in urethane (1 g/kg, i.p.). The auditory nerve was exposed via a craniotomy, partial aspiration of the cerebellum, and placement of small cotton balls to separate the cochlear nucleus from the temporal bone. The bulla was widely open to visualize the apical end of the cochlea.

### Acoustic stimulation

Sound was delivered by coupling the sound source, a Bayer DT-48 earphone, to the ear canal through a T-shaped plastic tube (Z105058, Sigma-Aldrich®). A metal probe of a ½" Brüel & Kjær (Brüel & Kjær, Nærum, Denmark) condenser microphone, which was inserted through the plastic tube, was used to measure the sound pressure within 2 mm of the tympanic membrane. For acoustic calibrations, single-tone stimuli were gated at onset and offset (5 ms rise/fall time). The duration of the tone pips was 100 ms. Stimulus frequency varied between 100 and 4000 Hz, in steps of 50 Hz. Three types of sound stimuli were used: clicks of brief duration, exponential swept-sine signals and Gaussian noise. Click stimuli (duration = 125 µs) were presented every 20 or 25 ms. Click levels are expressed as peak-equivalent SPL (dB pSPL) and were determined from middle-ear responses to tones: the pSPL of a click corresponds to the SPL of a 500 Hz tone with the same amplitude vibration. The exponential swept-sine waveform, or chirp, was generated according to the following formula ^11,12^:

1 $$x\left(t\right)=sin\left\{2\pi {f}_{1}L\left[\text{exp}\left(\frac{t}{L}\right)-1\right]\right\}$$

The coefficient *L* in Eq. (1) is defined as:

2 $$L=Tlog\left(\frac{{f}_{2}}{{f}_{1}}\right)$$

The duration of the chirp signal is *T* = 250 ms with initial (*f*_1_) and final frequencies (*f*_2_) equal to 100 and 1000 Hz, respectively. Chirp levels are expressed as dB SPL for a 500 Hz tone as determined from the acoustic calibration. Gaussian noise with a duration of 300 ms was generated using the Matlab function *randn* and was low-pass filtered at 3000 Hz before stimulus presentation. Noise levels are expressed in dB SPL using the acoustic calibration for a 500 Hz tone.

During the initial experiments, sound stimuli were generated using a 14-bit Analog Discovery sound card (Digilent, Pullman, Washington, USA). For later experiments, beginning with experiment OCT15, sounds were digitally generated using a 24-bit RME Fireface UC sound card (IMM Elektronik GmbH, Mittweida, Germany). Software to control the sound cards was written using Matlab® R2020b (Mathworks, Natick, Massachusetts, USA). In experiments conducted using the Analog Discovery card, the digital to analog (D/A) and analog to digital systems of the card started simultaneously. For experiments using the Fireface soundcard, the start of the D/A system was delayed by about 2.5 ms relative to the A/D.

### OCT vibrometry

Sound-evoked responses of the intact organ of Corti were recorded using a Telesto Spectral Domain OCT system (Thorlabs GmbH, Germany) and ThorImage® OCT version 5.4.2 (Thorlabs GmbH, Germany). Further analysis of the output of the ThorImage software was performed by ad-hoc programs written using Matlab® R2020b. Synchronization between the sound card and the Telesto system was achieved by enabling the external triggering option in ThorImage and a clock signal generated by the sound card. The frequency of the clock signal was modified using a custom-made frequency divider. The output rate of the sound card was usually 32 K samples/sec and the sampling rate of the Telesto system was set by the clock signal frequency at 8 K samples/sec.

### Data analysis

Direct impulse responses were obtained by presenting acoustic clicks and averaging the evoked vibrations of the OoC. Indirect impulse responses were obtained by presenting either the exponential swept-sine waveform (Eq. 1) or the broadband Gaussian noise. The indirect impulse response obtained using Eq. (1) was calculated via a deconvolution process^12^:

3 $$h\left(t\right)={F}^{-1}\left[\frac{F(y\left(t\right))}{F(x\left(t\right))}\right]$$

where $F$ and ${F}^{-1}$ represent the direct and inverse Fourier transform, respectively. $y(t)$ represents the sound-evoked vibration of the OoC at a given point.

The indirect impulse response was also computed by a cross-correlation between the noise stimulus, $x(t)$, and the sound-evoked vibration, $y\left(t\right)$, as follows^13^:

4 $$h\left( t \right) = \frac{1}{P}\varphi_{yx} \left( t \right)$$

The term $P$ above represents the power spectral density of the noise stimulus. The cross-correlation function, ${\varphi }_{yx}$, represents the inverse Fourier transform of the following term:

5 $$\emptyset_{yx} \left( {2\pi f} \right) = X^{*} \left( {2\pi f} \right)Y\left( {2\pi f} \right)$$

where ${\varnothing }_{yx}$, $X$, and $Y$ represent the Fourier transform of the ${\varphi }_{yx}(t)$, $x(t)$, and $y\left(t\right)$, respectively. The term $h(t)$ has been referred to as the first-order Wiener kernel in previous studies^13^. Each kernel presented here represents the average value of at least 40 kernels.

Fourier analysis was performed using the Matlab *fft* function. The analysis was performed on the complete waveforms after zero padding, except for the results shown in Fig. 5, where the waveforms were divided into two segments, as described in the Fig. 5 caption.

Cochlear responses to clicks were also modeled using discrete linear filters as detailed in^14^. Briefly, the fitting was performed using the Matlab *stmcb* function, which yields a transfer function with a finite number of zeros and poles. For OoC click responses the estimated filter is non-minimum phase, that is, some of the zeros of the transfer function were outside the unit circle. Minimum-phase approximations were obtained by replacing the zeros outside the unit circle ($\left|{z}_{i}\right|>1$) by the inverse of their complex conjugates. In other words, all zeros ${z}_{i}$ outside the unit circle were replaced by $\frac{1}{{z}_{i}^{*}}$ . The model of the cochlear impulse response consists of a frequency-independent delay plus the minimum-phase approximation ($delay+mp$). The delay duration was set to maximize the best fit of the original click response.

### Ethical approval

Experiments were performed at the University of Castilla-La Mancha, School of Medicine in Albacete, Spain. All procedures were approved by the Institutional Animal Care and Use Committee of the University of Castilla-La Mancha (protocol: PR-2020-02-06) in accordance with the relevant guidelines and regulations including the ARRIVE guidelines.

## Data Availability

Data used in this study are available from the corresponding author upon reasonable request.
